# Mitochondrial Localization of Vitamin D Receptor in Human Platelets and Differentiated Megakaryocytes

**DOI:** 10.1371/journal.pone.0008670

**Published:** 2010-01-13

**Authors:** Francesca Silvagno, Enrico De Vivo, Angelo Attanasio, Valentina Gallo, Gianna Mazzucco, Gianpiero Pescarmona

**Affiliations:** 1 Department of Genetics, Biology and Biochemistry, University of Turin, Turin, Italy; 2 Center for Experimental Research and Medical Studies (CERMS), S. Giovanni Battista Hospital, Turin, Italy; 3 Department of Biomedical Sciences and Human Oncology, University of Turin, Turin, Italy; Instituto de Química, Universidade de São Paulo, Brazil

## Abstract

**Background:**

Like other steroid hormones, vitamin D elicits both transcriptional events and rapid non genomic effects. Vitamin D receptor (VDR) localization and mechanisms of VDR-triggered non genomic responses are still controversial. Although anticoagulant effects of vitamin D have been reported and VDR signalling has been characterized in monocytes and vascular cells, nothing is known about VDR expression and functions in human platelets, anucleated fragments of megakaryocytes which are known targets of other steroids.

**Methodology/Principal Findings:**

In this study we characterized the expression and cellular localization of VDR in human platelets and in a megakaryocyte lineage. Human platelets and their TPA-differentiated precursors expressed a classical 50 kDa VDR protein, which increased with megakaryocytes maturation. By biochemical fractionation studies we demonstrated the presence of the receptor in the soluble and mitochondrial compartment of human platelets, and the observation was confirmed by immunoelectron microscopy analysis. Similar localization was found in mature megakaryocytes, where besides its classical nuclear localization the receptor was evident as soluble and mitochondria resident protein.

**Conclusions:**

The results reported here suggest that megakaryocytopoiesis and platelet activation, which are calcium-dependent events, might be modulated by a mitochondrial non genomic activity of VDR. These data open challenging future studies on VDR physiological role in platelets and more generally in mitochondria.

## Introduction

The vitamin D endocrine system plays a crucial role in calcium homeostasis and bone metabolism [1], [2]. The biological effects of 1,25(OH)_2_D_3_ are mediated mainly by its interaction with the vitamin D receptor (VDR), which belongs to the same family as the steroid and retinoid receptors [3], via genomic and non genomic mechanisms of action. In fact besides the classical VDR role as transcription factor vitamin D compounds, like other steroid hormones, can also elicit responses that are too rapid to involve changes in gene expression and appear to be mediated by cell surface receptors. Among the non-genomic actions of 1a,25-dihydroxy vitamin D3 are the opening of L-type Ca2þ channels in osteoblasts which results in a rapid increase of intracellular calcium [4]. The extranuclear receptor localization is still controversial. Several reports indicate a subcellular distribution in the cytoplasm, in discrete regions of the nucleus and along the nuclear envelope [5], whereas the membrane-initiated effects are attributed to a plasma membrane-associated receptor [6]; in fact VDR has been found in cavolae-enriched plasma membrane [7]. Moreover microscopy studies have revealed that VDR has mitochondrial, membrane, cytosol and perinuclear localization [8]. During the past two decades an increasing number of experimental data have revealed a broad range of biological actions for VDR, that include induction of cell differentiation [9], [10], inhibition of cell growth [11], immuno-modulation [12], [13], and control of other hormonal systems [14], [15]. In addition to vitamin D classical target tissues, VDR is also expressed in monocytic cells [16] and vascular endothelial cells [17], suggesting potential roles of vitamin D in antithrombotic functions. It has been demonstrated the anticoagulant effects of vitamin D in terms of up-regulation of thrombomodulin and down-regulation of coagulation tissue factor in monocytes [16], [18] and in vivo in aorta, liver and kidney [19]. While it is clear that the VDR/vitamin D system plays an important role in maintaining normal antithrombotic homeostasis in vivo, nothing is known about VDR expression and function in platelets, the main players in thrombus formation. Platelets are anucleated fragments of megacaryocytes whose maturation and aggregation is calcium-driven and therefore potentially modulated by a non genomic activity of VDR. The major structural features of megakaryocytic differentiation are an increase in nuclear size with DNA polyploidization and an increase in cytoplasmic volume with formation of secretory granules and demarcation membranes. Cytoplasmic fragments rich in mitochondria are then released and form proplatelets. These structural changes are accompanied by progressive expression of adhesive glycoprotein complexes implicated in platelet function and by increases in Ca^2+^ mobilization and Ca^2+^ influx by the G_q_-coupled receptor agonists, thrombin and thromboxane A_2_ [20]. The aim of this work was to evaluate the expression of VDR in human platelets and characterize its intracellular localization in order to suggest a physiological role of the receptor. We found that human platelets express VDR, which is mainly located in the mitochondrial compartment. Moreover VDR expression is enhanced during differentiation of a megakaryocyte cell line, suggesting the requirement of VDR signalling in mature platelets.

## Materials and Methods

### Primary Antibodies

The following antibodies against VDR were used: rabbit polyclonal anti-VDR (C-terminus fragment) clone C-20 (sc-1008, Santa Cruz Biotechnology, CA); rat monoclonal anti-VDR biotin-labelled (aa 89-105 epitope) clone 9A7γ.E10.E4 (RT-200-B, LabVision NeoMarkers, CA). Polyclonal antibody against GAPDH and monoclonal antibodies against CD34, CD41 and CD42b were from Santa Cruz. Polyclonal antibody anti-COX-1 was from Cayman-Chemical Co, Ann Arbor, MI. Monoclonal antibody anti-porin (31HL) was purchased from Calbiochem, La Jolla, CA. Polyclonal antibody against Von Willebrand Factor was obtained from Sigma.

### Platelets Isolation

Peripheral blood samples were collected with written informed consent from blood donations by healthy adult donors of both sexes provided by the local blood bank (S. Giovanni Battista Hospital, Turin, Italy). Samples were collected into sterile acid citrate dextrose anticoagulant and centrifuged twice at 200 x g for 15 min; the resultant platelet-rich plasma (PRP) was used as source of platelets. Platelets were isolated by centrifugation of PRP at 1,400 x g for 10 min, and recovered after two washes in washing solution 1 (12.72 mM sodium citrate, 2.99 mM glucose, 9.41 mM NaCl, 0.55 mM EDTA) and washing solution 2 (1 mM Tris-HCl, 2.99 mM glucose, 15.38 mM NaCl, 0.55 mM EDTA, pH 7.4).

### Cell Culture and Flow Cytometry Analysis

The human megakaryoblastic leukaemia cell line MEG-01 was purchased from ATCC (Rockville, MD), cultured in in RPMI-1640 with Glutamax (Invitrogen), 10% foetal bovine serum and 1% penicillin (100 IU/ml)/streptomycin (100 µg/ml), and maintained in plastic dishes in a humidified atmosphere at 37°C and 5% CO_2_. Cells were treated for reported time with 16 nM TPA and medium changed every four days. Indirect immunofluorescent tests were performed by incubating 0.5x10^6^ cells in suspension with purified monoclonal antibodies (dil. 1∶200 in PBS + 1% heat-inactivated fetal bovine serum, FBS) for 30 min at 4°C. Bound antibodies were revealed by fluorescein isothiocyanate (FITC)-conjugated F(ab’)_2_ sheep anti-mouse IgG (dil. 1∶200, Sigma Aldrich, Inc., St. Louis, MO). Cells were then analyzed on a FACScan flow cytometer (Becton Dickinson) by CellQuest software.

### Extracts Preparation, Immunoprecipitation and Western Blotting

Platelet and MEG-01 lysates for western blotting were prepared by a hot lysis method, carried out resuspending the samples in boiling sample buffer 1x (SB1x: 100 mM Tris-HCl pH 6.8, 15% glycerol, 2% SDS, and 1% protease inhibitor cocktail set III (Calbiochem-Novabiochem Corporation, La Jolla, CA). For immunoprecipitation studies platelets were resuspended in lysis buffer (25 mM Hepes pH 7.4, 135 mM NaCl, 1% NP40, 5 mM EDTA, 1 mM EGTA, 50 mM NaF, 10% glycerol and 1% protease inhibitor cocktail set III) and incubated at 4°C for 15 min with constant rotation. Lysates (500 µg of proteins) were then incubated with rotation overnight at 4°C with 2 µg of rabbit polyclonal antibody anti-VDR, followed by an additional incubation for 2 h with protein G agarose beads. Proteins were collected by centrifugation after boiling in SB 1x. Total cell lysates (30 µg) and immune complexes were separated by 10% SDS-PAGE and then transferred overnight to PVDF membranes (Immobilon-P, Millipore, Bedford, MA). Western blotting analysis was performed as described previously [21]. Proteins were immunostained with the indicated primary antibodies for 1 h at room temperature. Detection of protein of interest was performed using peroxidase-conjugated secondary antibodies (Pierce, Rockford, IL) followed by ECL detection (ECL detection kit, Perkin Elmer Life Science, Boston, MA, USA). Identification of VDR from immunoprecipitates was carried out with the biotinylated 9A7γ primary antibody followed by a streptavidin-HRP incubation. VDR bands from protein electrophoresis were quantified by scanning digital densitometry using an ImageJ software analysis (Sun Microsystems Inc., Palo Alto, CA); each VDR band was normalized by detection and densitometric analysis of a housekeeping gene (GAPDH).

### Purification of Caveolae

A detergent-free method for purifying caveolae membrane was published by Smart et al [22], and was followed with some modifications. 100 ml of PRP were the source of a platelets pellet, which was resuspended in hypotonic lysis buffer (10 mM Tris-HCl, pH 7.4 and 1% protease inhibitor cocktail set III). After incubation for 30 min on ice platelets were lysed by five passages through an insulin syringe needle. Samples were spinned for 10 min at 1000 x g and pellets were subjected again to the same lysis procedure. The two supernatants were combined and an aliquot kept for protein analysis (total extract). Samples were layered on top of 30% Percoll and centrifuged at 84,000 x g for 30 min in a Beckman SW40 Ti rotor. The plasma membrane fraction, a visible band at 2/3 from the bottom of the centrifuge tube, was collected and sonicated twice on ice. An aliquot of the sonicate was saved (plasma membrane, PM) before separating the remainder on a linear 20% to 10% OptiPrep gradient by centrifugation at 52,000 x g for 90 min in a Beckman SW40 Ti rotor. The top 5 ml of the gradient was collected, was overlaid with 5% OptiPrep and centrifuged at 52,000 x g for 90 min in a Beckman SW40 Ti rotor. A distinct opaque band present at the overlay interface was collected and designated caveolar membranes. Proteins were pelleted by ultracentrifugation at 100,000 x g for 60 min in a Beckman 70.1 Ti rotor and resuspended in 50 µl of SB 1x.

### Purification of Alpha Granules and Mitochondria

Subcellular fractionation was carried out following the procedure described by Broekman et al [23], with some modifications. Total extract was obtained from 350 ml of PRP as described for caveolae purification and an aliquot kept for protein analysis. One fifth of total extract was centrifuged at 100,000 x g for 60 min in a Beckman 70.1 Ti rotor. Pellet was resuspended in 50 µl of sample buffer 1x and designated total membranes, while supernatant represented the soluble fraction of platelets. The remainder of total extract was layered on linear 30–60% sucrose gradient and centrifuged at 134,000 x g for 120 min in a Beckman SW40 Ti rotor. Particulate zones were identified by their light-scattering properties, and after harvesting were diluted three- to fivefold with 0 .29 M sucrose and pelleted by ultracentrifugation at 100,000 x g for 60 min in a Beckman 70.1 Ti rotor before resuspension in 50 µl of sample buffer 1x. A typical density gradient separation showed two mainly visible bands, further characterized in their mitochondria or alpha granules content by the presence of organelles markers.

### Proteinase K Treatment

Mitochondria isolated by differential centrifugation at 10,000 x g for 10 min were treated with proteinase K (Sigma) at a final concentration of 50 µg/ml for 25 min at 0°C. Phenylmethylsulfonyl fluoride was then added to a final concentration of 2 mM, and samples were incubated for a further 10 min at 0°C. Mitochondria were then pelleted by centrifugation, lysed in 50 µl of SB 1x and equal amount of proteins analysed by western blotting.

### Immunoelectron Microscopy

Platelets were isolated from PRP as described in platelets isolation section. MEG-01 cells were collected by trypsinization. Both platelets and MEG-01 cells were prepared for immunoelectron microscopy as previously described [24]. Briefly, after fixation samples were incubated with polyclonal rabbit anti-VDR (200ug/ml, dil.1∶20), overnight at 4°C, followed by incubation for 1 h with GAR 15 (gold conjugated anti-rabbit antibody, 15 nm diameter, British Biocell International, Cardiff UK). Negative controls were performed by omitting the primary antibody. Samples were examined with a Philips CM 10 electron microscope (Philips, Eindoven, The Netherlands) after uranyl acetate and lead citrate staining.

### Statistical Analysis

Statistical analysis of data was performed using one-way ANOVA test with Tuckey correction. *p* values <0.05 were considered significant and indicated. All experiments were repeated at least 3 times and quantitative data were expressed as mean ± S.E.M or as mean ± S.D.

## Results

### Human Platelets Express VDR

**Figure 1:**
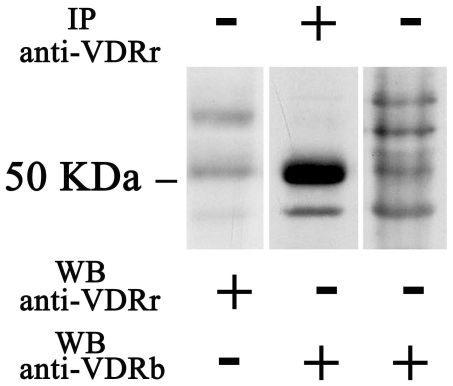
VDR expression in human platelets extracts. 30 µg of proteins from whole lysates were analysed for VDR expression by western blotting using two different antibodies. Left panel: whole lysate was evaluated by rabbit anti-VDR antibody (anti-VDRr). Right panel: VDR from whole lysate was detected by monoclonal biotinylated antibody (anti-VDRb). Middle panel: lysate was immunoprecipitate with anti-VDRr and detection by western blotting was performed with anti-VDRb. The 50 KDa molecular weight is shown for reference.

Western blot analysis of whole lysate from human platelets was performed with two antibodies raised against different regions of the VDR protein. Both the rabbit polyclonal and the monoclonal 9A7 gamma anti-VDR showed an immunoreactive band at the reported molecular weight of about 50 kDa [8], [25], together with several non specific bands (Figure 1, left and right lane). The identity of the 50 kDa VDR protein was proved by immunoprecipitation studies, which confirmed the recognition of the same protein by the two antibodies (Figure 1, middle lane). Immunoprecipitation was performed using the rabbit polyclonal anti-VDR antibody and detection was carried out with the biotinylated 9A7γ primary antibody followed by a streptavidin-HRP incubation, in order to avoid the signal given by immunoglobulins. The specificity of the two antibodies for different epitopes allowed the elimination of non specific bands and showed a prominent signal at the correct molecular weight. Further studies on VDR expression and localization were performed using the polyclonal antibody, which gave the clearest 50 kDa signal.

### Platelet VDR Is Not a Plasma Membrane Protein and Is Mainly Localized in the Mitochondrial Subcellular Fraction

**Figure 2:**
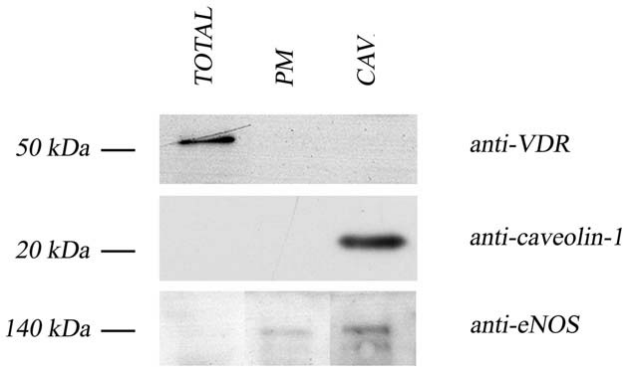
VDR expression on human platelets plasma membrane and caveolae. After subcellular fractionation procedures described in methods, 10 µg of proteins from total extracts (TOTAL), plasma membrane fraction (PM) and caveolae fraction (CAV) were separated by SDS-PAGE and analysed for VDR expression by western blotting using the rabbit anti-VDR antibody (upper panel). To validate the soundness of purification, the enrichment in two caveolae-associated proteins was checked by detection of caveolin (middle panel) and endothelial NOS (lower panel) with the corresponding antibodies. Reference molecular weights are shown on left.

The anucleated platelets are a good model to study the extranuclear VDR localization involved in the non genomic response to vitamin D. We decided to characterize the subcellular distribution of the receptor by biochemical protein fractionation studies. We evaluated first its possible expression on plasma membrane, since VDR has been found in the caveolae-enriched membrane regions of several cells [7], [26]. Plasma membranes and caveolae fraction were isolated from whole lysate by a detergent-free purification protocol based on gradient fractionation, and equal amounts of proteins were analysed by western blotting. As shown in figure 2 VDR expression, evident in whole cell lysate, was absent in both plasma membrane and caveolae fraction. To validate the soundness of our purification we checked the content of two proteins known as resident in the caveolar regions of the plasma membrane: caveolin resulted highly concentrated in caveolar fraction and endothelial NOS was gradually enriched from whole lysate to plasma membrane and even more in caveolae.

**Figure 3:**
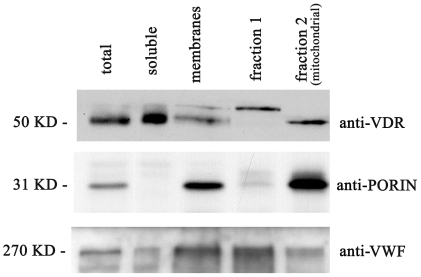
VDR distribution in soluble and mitochondrial fractions of human platelets. Following density gradient purification and ultracentrifugation as described in methods, subcellular fractions were obtained and 30 µg of each sample was analysed by western blotting. VDR rabbit antibody detected a 50 KDa band in total extract (total), cytosolic fraction (soluble) and total membranes preparation (membranes). VDR expression was enriched in fraction 2 (mitochondrial) while was absent in fraction 1. To assess the composition of fractions, the detection of a mitochondrial protein (porin) and an alpha-granules associated protein (Von Willebrand Factor VWF) was performed. Porin resulted enriched in membranes fraction and markedly in mitochondrial fraction 1, while VWF was mainly found in fraction 1. Reference molecular weights are shown on left.

We next checked whether platelet VDR was a soluble cytoplasmic protein or associated with the mitochondrial compartment, particularly abundant in platelets. The latter subcellular localization was suggested by recent works which found the receptor in the mitochondria of rat intestinal cells [8]. Proteins from whole lysate were subjected to ultracentrifugation to obtain a particulate fraction comprehensive of all proteins associated to membranes or vesicles and a soluble fraction containing the cytoplasmatic proteins. Protein analysis of the soluble and particulate fraction (Figure 3) revealed a VDR both soluble and membrane associated. Moreover from the whole lysate upon density gradient centrifugation we purified a fraction highly enriched in mitochondria and a fraction mainly composed by alpha granules. We analysed VDR expression in all fractions by western blotting and we assessed the content of all fractions in terms of porin as mitochondrial marker and Von Willebrand Factor (VWF) as protein associated to alpha granules. As described in methods and showed in Figure 3, we obtained a fraction 1 mainly composed by alpha granules (VWF positive and scarce porin content) which did not show VDR expression. From the same purification procedure we extracted a fraction 2 mainly mitochondrial in nature (porin positive with minor content of VWF, a contamination found also in the soluble fraction) which was highly enriched in VDR. From this biochemical study we could conclude that platelet VDR is substantially associated with the mitochondrial compartment.

### VDR Is Stored in Mitochondria of Human Platelets

**Figure 4:**
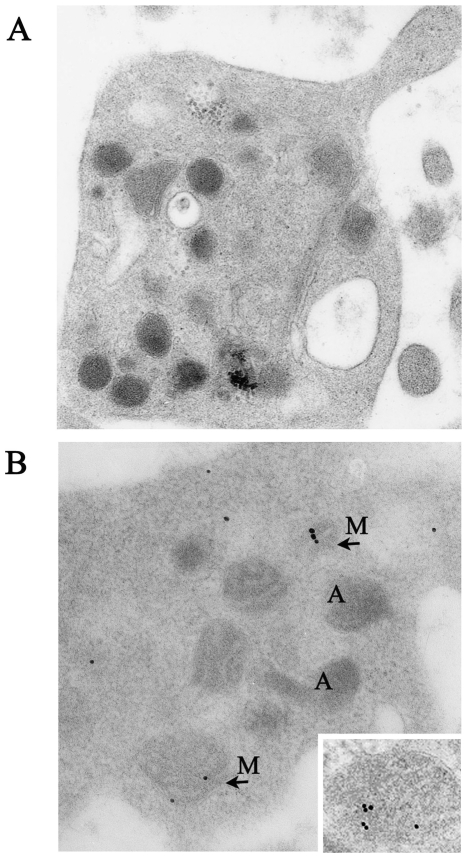
The VDR storage site in human platelets. (A) Platelets observation by electron microscopy revealed a well preserved cell structure and organization of intracellular granules. Sample was processed in absence of anti-VDR primary antibody. (B) Immunogold labelling of VDR in human platelets, showing occasional VDR labelling in mitochondria (M) and cytosol, with no significant labelling of alpha granules (A), or plasma membrane. Inset shows a mitochondrial structure under higher magnification. Experiments were performed as outlined in Immunoelectron microscopy section.

Immunoelectron microscopy of platelets was performed to confirm the conclusions drawn from the biochemical fractionation studies. As shown in figure 4A, platelet structures were well preserved after immunogold labeling procedures. In agreement with the intracellular distribution suggested by western blotting analysis, the anti-VDR antibody revealed the presence of the receptor in the mitochondrial structures and in the cytosol, without significant labeling of other platelet structures (Figure 4B).

### VDR Expression Increases during Megakaryocyte Differentiation

**Figure 5:**
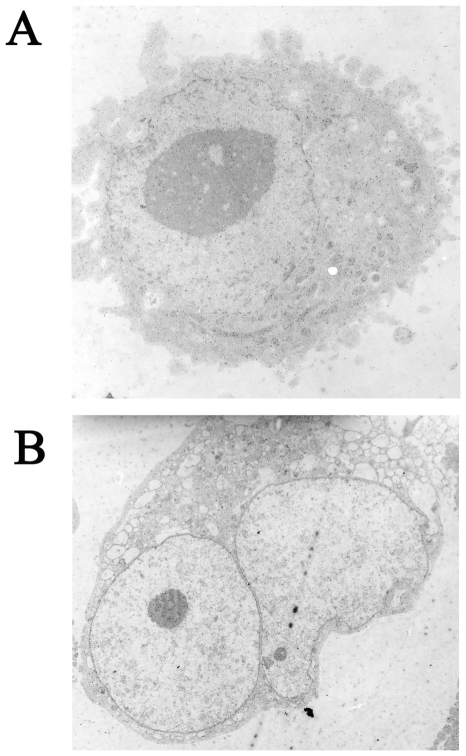
Morphological changes of MEG-01 cells after TPA-promoted differentiation. (A) Electron microscopic photograph of control MEG-0l cells. The round nucleus and the relatively developed cytosol with homogeneous organization can be observed. (B) Electron microscopic photograph of MEG-0l cells treated for eight days with 16 nM TPA. Peripherally located and multiple nuclei are evident. Prominent cytoplasmic blebs are observed.

**Figure 6:**
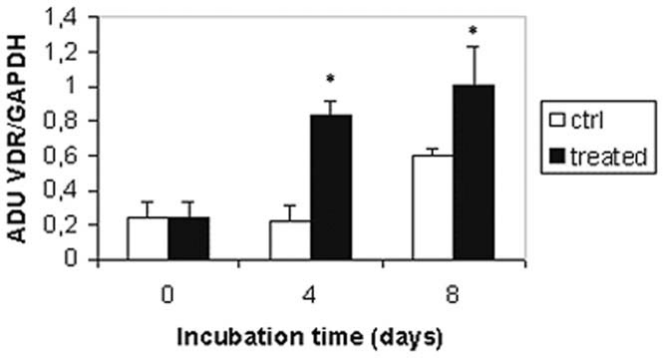
VDR expression during megakaryocyte maturation triggered by TPA. MEG-01 cells were incubated in absence or presence of 16 nM TPA and harvested at the indicated times. 50 µg of total lysate were analysed by western blotting using the rabbit anti-VDR antibody and an anti-GAPDH antibody. VDR bands from four independent experiments were quantified, normalized for loading as a ratio to GAPDH expression and data plotted on graph as arbitrary density units. Data represent the mean ± S.E.M. *p<0.05 compared to control at time zero.

Platelets are derived from megakaryocytes resident in the bone marrow. During megakaryocyte maturation, the polyploid cell undergoes a complex process of cytoskeletal rearrangement, followed by proplatelet elongation, and the release of cytoplasmic fragments as circulating platelets [27]. Since platelets are anucleated and contain only a trivial amount of mRNA, they lack significant capacity for protein synthesis and regulation of vitamin D receptor protein expression must occur during megakaryocytopoiesis or thrombopoiesis. It has been reported that VDR mRNA is expressed in megakaryoblastic cells and the treatment with forskolin, a differentiating drug activator of adenylate cyclase, causes an increase in VDR mRNA levels [28]. In order to test VDR modulation during the differentiative process, we analysed receptor expression in MEG-01 cells, a human megakaryoblastic leukaemia cell line which maintains some characteristics of megakaryocytes. Differentiation was triggered in vitro by the phorbolester 12-*O*-tetradecanoylphorbol-13-acetate (TPA), a stimulator of protein kinase C known to induce megakaryocytic maturation as demonstrated by enlargement of cell size, multiplication of nuclei, increase in granularity and in expression of platelet-specific proteins [29], [30]. MEG-01 cells were treated for eight days with 16 nM TPA and then their differentiated state was characterized in terms of morphological changes and protein pattern expression. A prominent response to TPA treatment was demonstrated as shown in figure 5: the cells became weakly adherent and displayed mature megakaryocytic characteristics by electron microscopy such as the development of cytoplasmic blebs and a multiplication of nuclei (figure 5B). MEG-01 cells were periodically harvested during incubation in absence (control: C0, C4 and C8) and in presence of TPA (T4 and T8). The most used surface antigens markers of early myeloid (CD34) and mature megakaryocyte stage (CD41, CD42b) [31], [32] were evaluated by flow cytometry analysis. Moreover we quantified by western blotting the expression of the enzyme COX-1, since it has been reported that it increases consistently over the full course of MEG-01 differentiation [33]. The results of our evaluation of TPA-induced differentiated state of MEG-01 are summarized in table 1. We observed a decrease of CD34 antigen and an increase of CD41 and CD42 antigen expression during differentiation (fourth day, T4) and even more at the end of eight days of TPA treatment (T8). Similarly, COX-1 content of differentiated MEG-01 consistently increased. Untreated cells did not show any variation in the content of all markers (C4 and C8). TPA-treated MEG-01 cells represent therefore an attractive model to study the expression of VDR during differentiation of megakaryocytes. Such expression was evaluated by western blotting in the same experimental conditions. The 50 kDa VDR band was quantified and results expressed in arbitrary density units were plotted on graph as shown in figure 6. Interestingly, VDR content was enhanced along the differentiation process; in fact the receptor, fairly expressed in control cells (C0), was more abundant after four days of treatment (T4) and at the end of the maturation process (T8), whereas untreated cells did not show a significant increase of VDR over the same period of incubation (C8).

**Table 1:** Analysis of megakaryocyte maturation markers.

	CD34	CD41	CD42	COX-1
**C0**	12,73±0,257	1,15±0,043	1,03±0,03	0,12±0,009
**C4**	11,09±2,054	1,23±0,147	1,03±0,036	0,12±0,022
**C8**	9,35±1,058 *	1,48±0,342	1,06±0,154	0,16±0,03
**T4**	2,95±1,749 ** °	2,31±0,856 * °	1,89±0,112 * °	0,59±0,031 * °
**T8**	2,69±1,749 ** ^	3,02±0,58 ** ^	2,41±0,742 ** ^	0,69±0,037 * ^ §

* : p<0,05 vs C0; *: p<0,05 vs C0; *: p<0,05 vs C0; *: p<0,0001 vs C0.

** : p<0,0001 vs C0; **: p<0,001 vs C0; **: p<0,0001 vs C0; °: p<0,0001 vs C4.

°: p<0,0001 vs C4; °: p<0,05 vs C4; °: p<0,05 vs C4; ^: p<0,0001 vs C8.

^: p<0,0001 vs C8; ^: p<0,01 vs C8; ^: p<0,0001 vs C8; §: p<0,001 vs T4.

MEG-01 cells were analysed for their maturation stage after four (T4) and eight (T8) days of treatment with 16 nM TPA and compared with control cells at the same incubation time (C0, C4, C8). Markers of early myeloid (CD34) and mature megakaryocyte stage (CD41, CD42b) were evaluated by flow cytometry analysis, whereas the expression of COX-1 was assessed by western blotting analysis and quantification of immunoreactive bands normalized as a ratio to GAPDH expression. Data from three independent experiments were used and are shown as mean ± S.D. Significance of data referred to treated vs control cells is indicated at the bottom.

### Megakaryocyte VDR Is Distributed in Nuclear, Cytosolic and Mitochondrial Compartments

**Figure 7:**
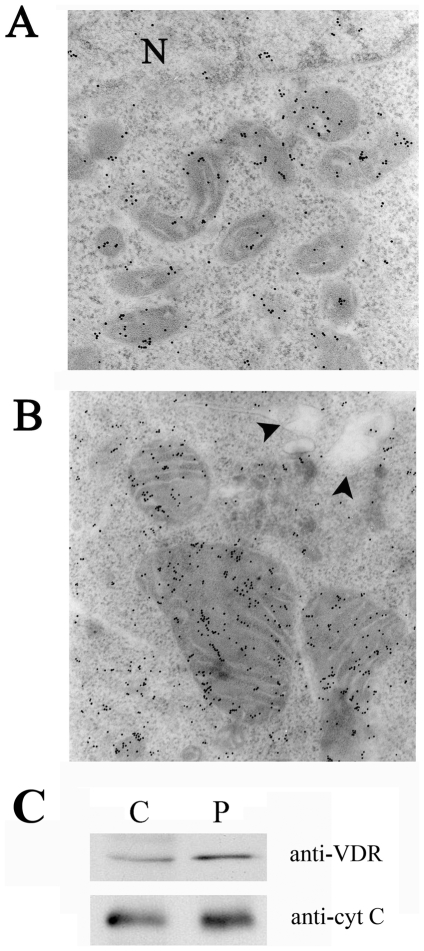
VDR distribution in intracellular compartments of MEG-01 cells. (A) Electron micrograph of control MEG-0l cells reacted with rabbit anti-VDR antibody. Nuclear space (N), cytosol and mitochondria are positive for VDR. (B) Electron micrograph of MEG-01 cells treated for eight days with 16 nM TPA reacted with anti-VDR antibody. Mitochondria and cytosolic areas are positive for VDR. Cytoplasmic blebs are evident (closed arrowheads). (C) Treatment of mitochondria from MEG-01 cells with proteinase K. Proteins from mitochondria untreated (control, C) or treated with proteinase K (P) were analysed by western blotting using antibodies against VDR and cytochrome C.

The same immunoelectron microscopy analysis performed on platelets was carried out on MEG-01 cells. As shown in figure 7A untreated cell were positive for VDR labeling and displayed a receptor expressed in the nuclear region, cytosol and most interestingly in mitochondrial organelles. The same pattern was evident in TPA-differentiated cells, with a more evident presence in mitochondria (figure 7B). Microscopy observations confirm therefore the data collected by protein analysis, and reveal a mitochondrial localization of VDR even in megakaryocytes, the platelets precursor cells. Even though the microscopy images clearly showed a VDR localized in the inner compartment of mitochondria, in order to definitely exclude that the receptor is attached to organelles and not incorporated, a treatment with proteinase K was performed on mitochondrial extracts. The levels of VDR and cytochrome C, another internal mitochondrial protein, were not significantly affected by the treatment, as shown in figure 7C.

## Discussion

In this work we report for the first time not only the presence of VDR in human platelets but also most interestingly its mitochondrial localization. Like for other tissues, non genomic effects of VDR have been documented in vascular and blood cells too. The most known rapid non genomic effects of vitamin D are associated to the control of intracellular calcium levels. In fact 1,25(OH)_2_D_3_ can rapidly stimulate phosphoinositide metabolism, cytosolic calcium levels, cGMP levels, PKC, MAP kinases, and the opening of chloride channels (reviewed in [34]). In human peripheral blood mononuclear cells vitamin D is capable of exerting a rapid non genomic effect on intracellular calcium concentration [35]. 1,25(OH)_2_D_3_ induces vascular smooth muscle cell (VSMC) migration independent of gene transcription via PI3 kinase pathway [36]. Up to now nothing was known about VDR expression in platelets. The idea that these anucleated cells are responsive to the obviously non genomic effects of steroids is not new. Several evidences indicate that sex hormones affect platelet physiology, and it has been reported the expression of estrogen receptor (ER) beta and androgen receptor (AR) in human platelets [37]. Moreover it has been described the existence of glucocorticoid receptor (GR)/mineralcorticoid receptor (MR) heterodimers in platelets [38]. Our work demonstrates the presence of a classical form of VDR in human platelets. When we sought to investigate its subcellular distribution, we hypothesized a similarity with ER beta localization in caveolar structures of platelets plasmamembrane, a binding which has been demonstrated important for its intracellular signalling [39]. Several findings support the notion that rapid estrogen signaling is mediated by ER associated with specialized lipid raft domains that serve to concentrate and provide a matrix for receptors and related signaling proteins [40], [41]. However, by a purification procedure based on gradient centrifugation we discovered that platelets plasma membrane and caveolar fraction resulted devoid of the receptor. A soluble protein was hence expected, since the cytosolic localization has been reported for ER beta and AR in platelets [37]; nevertheless a possible alternative was suggested by a recent work of Gonzalez-Pardo and coll., who described a mitochondrial VDR [8]. Indeed, fractionation studies demonstrated the presence of the receptor in the mitochondrial compartment, and the observation was confirmed by immunoelectron microscopy analysis of platelets. These data open challenging future studies on VDR physiological role in platelets and more generally in mitochondria. Further investigations are needed in order to understand the mechanisms of VDR mitochondrial import and its function in these organelles. The intracellular distribution of VDR is dependent not only on subcellular localization signals encoded in the protein but also on the nature and composition of the multi-molecular complexes formed by the receptor. A mitochondrial localization signal has not been described yet for VDR protein, however future analysis could show a putative internal mitochondrial targeting polypeptide signal (mtTPs) similarly to the one identified in human ERβ [42] and in glucocorticoid receptor [43], which are localized in mitochondria. Alternatively VDR association with other molecules could facilitate its translocation. It has been shown that post-translational events substantially alter RXRα location by addressing the receptor to mitochondria, where it binds to mitochondrial DNA by forming heterodimerical complexes with p43, a truncated version of the triiodothyronine nuclear receptor [44]. Since RXR is a VDR binding partner one could reasonably postulate a heterodimeric RXR/VDR mitochondrial translocation and transcriptional activity. Besides the transcriptional control of mitochondrial genes steroid hormones may influence mitochondrial function by non genomic interactions that may control mitochondrial cation fluxes (reviewed in [45]). These actions regulate major mitochondrial functions including mitochondrial-dependent apoptosis and the same role could be played by VDR in platelets.

Because platelets are anucleated, their protein content is a consequence of gene expression in precursor cells known as megakaryocytes. We used the immortalized human megakaryoblastic cell line MEG-01 as a model to study the expression and localization of VDR in platelets progenitors, since MEG-01 cells can be induced to differentiate into platelet-like structures by adding nanomolar concentrations of 12-0-tetradecanoylphorbol-13-acetate (TPA). In our study MEG-01 cells were able to differentiate morphologically and biochemically into more mature megakaryocytes and platelet-like structures after stimulation with phorbol esters and, therefore, represent an attractive model to study the modulation of VDR expression during differentiation of megakaryocytes. As reasonably expected, VDR expression and localization found in human platelets was matched by the presence of the receptor in precursor cells, a presence which is reinforced during a differentiation process which preludes to platelets formation. Likewise platelets analysis, to our knowledge this is the first report characterizing VDR expression and localization in megakaryocytes. It has been described the expression of other steroid hormone receptors in human megakaryocytes and their increase during differentiation [37]. On the other hand few published data demonstrate that the megakaryocyte differentiation process can be triggered by estrogens, vitamin D, and glucocorticoids [28], [46], [47]. The differentiative properties of vitamin D are augmented by forskolin, an activator of adenylate cyclase and modulator of cell proliferation and differentiation [28]. These observations suggest that 1,25(OH)_2_D_3_ might play an important role in megakaryocytopoiesis or at least potentiate the effect of other antiproliferative stimuli.

The role of VDR in progenitors differentiation and mature platelets remains to be elucidated. The control of calcium homeostasis is the most probable nongenomic function of platelet VDR, given the important role of calcium fluxes in platelets formation, aggregation and granule content release. This possibility is supported by observations on nongenomic effects of sex hormones in platelets [48], such as the fact that in vitro testosterone appears to enhance platelet aggregation [37]. As modulator of calcium fluxes VDR could play an essential role in megakaryocytopoiesis, platelets activation and apoptosis, which are calcium-dependent events [20], [49].
